# Workforce planning and development in times of delivery system transformation

**DOI:** 10.1186/s12960-016-0154-3

**Published:** 2016-09-23

**Authors:** Patricia Pittman, Ellen Scully-Russ

**Affiliations:** 1 Milken Institute School of Public Health, The George Washington University, 2175 K Street, NW, Suite 500, Washington, DC, 20037 United States of America; 2 Graduate School of Education and Human Development, The George Washington University, 2136 G Street, NW, Washington, DC, 20052 United States of America

**Keywords:** Workforce planning and development, Human resources in health, Healthcare delivery reform, System change, Loosely coupled systems, Labor-management partnerships, US Affordable Care Act

## Abstract

**Background:**

As implementation of the US Affordable Care Act (ACA) advances, many domestic health systems are considering major changes in how the healthcare workforce is organized. The purpose of this study is to explore the dynamic processes and interactions by which workforce planning and development (WFPD) is evolving in this new environment.

**Methods:**

Informed by the theory of loosely coupled systems (LCS), we use a case study design to examine how workforce changes are being managed in Kaiser Permanente and Montefiore Health System. We conducted site visits with in-depth interviews with 8 to 10 stakeholders in each organization.

**Results:**

Both systems demonstrate a concern for the impact of change on their workforce and have made commitments to avoid outsourcing and layoffs. Central workforce planning mechanisms have been replaced with strategies to integrate various stakeholders and units in alignment with strategic growth plans. Features of this new approach include early and continuous engagement of labor in innovation; the development of intermediary sense-making structures to garner resources, facilitate plans, and build consensus; and a whole system perspective, rather than a focus on single professions. We also identify seven principles underlying the WFPD processes in these two cases that can aid in development of a new and more adaptive workforce strategy in healthcare.

**Conclusions:**

Since passage of the ACA, healthcare systems are becoming larger and more complex. Insights from these case studies suggest that while organizational history and structure determined different areas of emphasis, our results indicate that large-scale system transformations in healthcare can be managed in ways that enhance the skills and capacities of the workforce. Our findings merit attention, not just by healthcare administrators and union leaders, but by policymakers and scholars interested in making WFPD policies at a state and national level more responsive.

## Background

As the implementation of the 2010 Affordable Care Act (ACA) advances in the United States, many healthcare organizations are taking bold measures to reorganize their delivery systems and finding that in order to do so, changes must be made to the healthcare workforce [1]. While different healthcare organizations in the United States, be they public or private, are at very different points in this process, commonly popular concepts include moving staff to new ambulatory and home care settings [2]; creating new jobs relating to care coordination and outreach to the sickest patients [3]; designing new modes of delivering care in response to consumerism [4]; adopting team-based care and task shifting based on the principal of practicing at the top of license and education [5]; requiring new roles and skills as part of the adoption of health information technologies (HIT); and the use of data for decision-making [6].

Understanding what workforce changes are occurring and how they are being managed is key not just for healthcare leaders but for policymakers as well. Traditional methods of projecting provider shortages and justifying the allocation of public funding to expand various professional pipelines are giving way to the notion that there are many models of care delivery and that they have vastly different staffing configurations. For example, several studies have demonstrated that including advanced practitioners in primary care medical homes allows practices to expand panel sizes [7, 8]. Choices about staffing, therefore, can have enormous implications for productivity, making assumption about the demand for certain health professions a moving target.

The policy question then becomes not just how will these changes alter the national demand for certain types of health workers at an aggregate level but how are organizations making choices about ways to reconfigure their workforce and, ultimately, what kinds of local, state, and federal policies are most supportive of workforce transformations that advance both workers’ well-being and the value of their services.

We know from the literature reviewing the hospital restructuring of the 1990s that workforce change management faces many challenges. The critiques of this era were many, but chief among them, according to Walston and colleagues, were the following: goals for change were not clear, too many changes were implemented too quickly, there was a lack of communication with employees, a lack of engagement with physicians and unions, there was a poor understanding of the local site differences by management leading to a one-size-fits-all approach, and, lastly, that training needs were not anticipated [9].

In a review of the international literature on workforce planning and development (WFPD), Curson and colleagues suggest that the problem goes deeper. They argue that workforce policies lack the capacity to respond to new demands for system change [10]. The reason, they point out, is that most workforce planning do not take account of political dynamics among the range of stakeholders outside the control of human resource administrators, be they at the organizational or the policy level.

It is with these critiques in mind that we are interested in understanding how two leading health systems in the United States, with a historic commitment to developing and retaining their workforce and to managing change through labor-management partnerships, are responding to the demands of the post-ACA environment. The aim is to explore how they are determining what changes are needed and how they are implementing those changes in practice. Their experiences may provide insights for other organizations, as well as for policymakers charged with ensuring that the healthcare workforce is able to meet population needs.

Our first case focuses on Kaiser Permanente (KP), an integrated system that has historically served the employer market on the West Coast. It has been at the forefront of systems that emphasize value over volume and among the organizations most advanced in the use of HIT to improve the patient care process. In addition, KP has one of the most successful models of labor-management partnerships (LMP) in the nation.

The second system is the Montefiore Health System, headquartered in the Bronx, NY, an organization with almost 20 years of experience with shared risk contracts with payers. Like KP, they have extensive experience with care coordination, they are in the process of expanding to new markets, and they have a LMP. They differ from KP in that their patient population is predominantly poor and Spanish speaking, and an extraordinary 80 % of their revenue is coming from Medicaid and Medicare.

### Conceptual framework

The objective of this study is to go beyond descriptive groupings of health workforce changes to explore the dynamic processes and interactions by which staffing models emerge. To frame our inquiry, we draw on the literature on health workforce planning and development and the theory of loosely coupled systems (LCS) [11].

For the purposes of this paper, we define WFPD as *the macro level processes and practices that enable the system to change and adopt new staffing arrangements and respond with timely and appropriate education, training, and certification programs*. Schrock has suggested that WFPD policies span the continuum of skill formation, employment networks, and career advancement [12]. This means not simply examining the supply and distribution of personnel in different categories but also understanding educational and training pathways, management of performance, and the regulation of working conditions.

Dussault and Dubois argue that the traditional approach to WFPD is a linear, sequential, and protracted skill formation process through which healthcare providers hand off demand projections to education institutions and certifying bodies that in turn, supply the requisite workforce [13]. Weick reasons that this form of sequential task interdependence induces rule-based action and cognitive processes that are not equipped to tackle ambiguous problems like providing a skilled workforce for care models that are in a constant state of flux [14]. This and other complex, non-routine problems require controlled cognition or slow, deliberative, and explicit thinking that is more often associated with reciprocal interdependence coordinated by an iterative process of negotiation and mutual adjustment among relatively autonomous units and subsystems. [14]

Dussault and Dubois describe an alternative approach that is emerging in healthcare that coordinates the efforts of a diverse range of institutional actors through adaptive processes that respond to specific, local political, economic, cultural, and social contexts where healthcare is delivered [13]. This approach is understood as a political exercise in which values and differences are made explicit, compromises are made, and actions are justified. Orton and Weick further suggest that there is a need to move beyond the traditional focus on static organizational elements, like structure, resource allocation, and technology, and turn instead to a focus on the dynamic relationship among them [15].

Organizational scholars developed the concept of “loose and tight coupling” as one way to examine complex organizational structures and relationships [16–19]. The focus of this approach is on hierarchy and interdependence among elements within and between organizations and how variability in these features enables different operational strategies and responses to shifts in the external environment [17]. In tightly coupled systems, individual units and organizations are linked together through formal structures and procedures and they respond to change through centralized control mechanisms that reduce variation and close the system off from the effects of external forces. In loosely coupled systems, on the other hand, the links among the components are weak and a high level of autonomy exists among the interdependent parts of the system [20]. While the variation in the way similar functions are organized and managed may make it difficult to integrate activities, theorists argue that it enables flexibility and openness to change in the environment [15].

According to the theory of LCS, all systems are both tightly and loosely coupled because there is variation in how subunits are linked and rely on each other (coupled)—as well as in the number and strength of their connections (lose or tight) [15, 17, 21]. Therefore, any subsystem may be closed to outside forces to ensure for stability (tight), while another subsystem may remain open to outside forces to enable flexibility (loose) [15].

This paradoxical nature of LCS makes it difficult for researchers to conceptualize and study [16], yet we would suggest that its application to the US healthcare system during this period of intense transformation holds explanatory potential. Healthcare systems are simultaneously being asked to expand coverage and access, while being financially incentivized to extend the continuum of care to address the social determinants and provide ongoing care management. As a result, there are significant pressures on traditional care models and staffing arrangements, leading in turn to the emergences of new patterns of “coupling,” both within and across healthcare organizations. Further, we submit that the effectiveness of the transformation occurring in healthcare today may hinge on new, more adaptive methods to prepare the healthcare workforce to perform in a more complex system of care, where job tasks, team interactions, and work locations are continuously changing.

To analyze changes in WFPD, we borrow from Weick’s typology of strategies for changing LCS [11] and from the descriptions on a new approach to WFPD in healthcare put forth by Curson et al. [10] and Dussault and Dubois [13] to identify a set of principles that together, may serve as a new adaptive WFPD framework aligned with the needs of a rapidly changing deliver system.

## Methods

We use a case study design to explore how two major health systems undergoing significant system transformation are managing the process of workforce change. We selected Kaiser Permanente (KP) and Montefiore because they are well known for their innovative approaches to integrating healthcare yet they are significantly different from each other with regard to their organizational histories, structures, and patient populations.

We conducted site visits to both organizations in the spring and summer of 2015, conducting interviews with 8–10 people at each site including executives, human resource managers, the heads of innovation and care coordination programs, and union and LMP representatives. Some interviews were held in group settings, while others were individual. We also conducted planning and follow-up phone calls with some of the participants. Interviews were taped and transcribed. We also reviewed current organizational documents, including training plans, reports, and collective bargaining agreements, as well as prior studies on each system [9, 22, 23].

Data analysis proceeded through several steps. First, the research team conducted a review of each case, including the historic development of the system and significant drivers of change, as well as the strategies, structures, and resources informants reported as being central to the competiveness of the system and the sustainability of the workforce in the post-ACA environment. To support this analysis, the research team developed a series of inductive and deductive codes, which we used to extract relevant data from the case documents and interview transcripts. Next, the researchers jointly analyzed the coded data to developed individual case profiles. These profiles were validated by key informants from each case. Finally, we conducted a constant comparative method to identify cross-cutting themes and principles to explain the workforce planning and development strategy emerging within the two systems.

## Results

### Case study 1: Kaiser Permanente

Kaiser Permanente (KP) was established in 1938 as a comprehensive medical system for the workers and their families at Kaiser steel mills and shipbuilding facilities across California and in Portland, OR. In 1945, after WWII ended and many shipyards closed, KP opened membership to the general public. The KP unions played an instrumental role in this expansion by helping KP market to unionized employers in areas where the company had a presence. Today, it operates as a Health Maintenance Organization (HMO) with 8.3 million health plan members in seven regions: Northern and Southern California, Colorado, Georgia, Hawaii, Mid-Atlantic, and the Northwest. Each region is made up of two separate entities, the Kaiser Foundation Health Plans and the Permanente Medical Group (PMG), a physician-owned corporation that owns and operates KP’s medical facilities. The PMG contracts with the Foundation to serve KP health plan members. A key feature in this model is that physicians are employed by KP. The national program office includes a variety of support functions, including human resources, labor relations, information technologies (IT), finance, and patient care services (nursing).

The KP Labor-Management Partnership (LMP) was formed in 1997. At the time, KP faced competitive pressures leading executives to demand deep union concessions. In response, many of the KP unions offered the company a choice: continued harsh labor-saving tactics and escalating labor strife, including a strike, or a partnership to address the fiscal crisis and improve the quality of care at KP. The company agreed to the partnership [24]. The governance structure consists of the LMP Strategy Group, with one representative from each of three sectors: Physicians, Management and Labor, and each region maintains its own tripartite LMP council.

By 2015, the LMP included 12 international and 28 local unions representing 105 000 KP employees or about half of the total KP workforce, across six of the seven regions. Hawaii is not part of the partnership, and not all KP unions are involved in the partnership, most notably absent is the California Nurses Association.

KP also has a network of functional units to support the design and management of change and WFPD strategies. The LMP staff is integrated into these units, and labor representatives are highly engaged in their activities. These units include the following:

- *National Workforce Planning and Development* (*housed in national human****r****esources* (*HR*)) provides opportunities to the KP workforce to optimize skills and competencies and manages two LMP education trusts: the Ben Hudnall Memorial Trust and SEIU/UHW Joint Employer Education Fund.
- *National Innovations Network including patient care services*, *workforce planning*, *and IT* functions as a loosely coupled “future-sensing” group that examines technology trends, creates proof of concepts and proof of technology, and develops pilots.
- *Unit-based teams* (*UBT*) are natural work groups of frontline workers, physicians, and managers who solve problems and enhance quality.

#### Drivers of change

KP’s history of pre-paid, member-based service is critical to understanding the company’s current competitive situation. KP is well positioned to grow in a post-ACA era in which policies to advance integration has proliferated. Growth has been especially dramatic in the Southern California Region, where new individuals that joined via the Health Exchange grew by 4 % per year (from 2 to 6 %). This rapid influx of new members has been most pronounced among younger and healthier individuals as compared to members in KP’s traditional employer-based plans.

KP leadership knew that they needed to understand the implications of this shift in demand and have held focus groups with their newest members. Results have led the company to reorient business strategy around three priorities, as follows:

1. *Convenience*. Millennials are demanding “care anywhere and how we want it.” Increased access, convenience, and enhanced experience of healthcare are therefore major priorities for the organizations.
2. *Affordability*. Because the individual market is more price sensitive than the group market, there is a heightened awareness that they must reduce the cost of care in order to continue to expand in this market.
3. *Value*. At the same time, new healthcare consumers expect more value or increased and enhanced services, and this is driving a number of efforts focused on the care experience.

#### Change strategies

Three strategic initiatives have emerged in response to these drivers. The LMP and the national innovation units are integrated into all three, as are KP members’ views, as represented through surveys, focus groups, and ethnographic studies.

- *Perform*, *Grow*, *Lead* is KP’s strategic plan. It emphasizes affordability targets, meeting rising customer expectations, and transforming care. Guiding principles include the following: *One KP*, which calls for a common care experience across all regions, and the *KP people strategy*, which articulates the desired characteristics of the KP workforce as “innovative, engaged, change ready, healthy, and accountable.”
- *Vision 2025* is an ongoing initiative to understand what healthcare consumers will look like and how KP can position itself to meet needs in a rapidly changing healthcare market. It develops care models and offers strategic road maps to guide planning and change. Health information technologies are central to this strategy, including the use of social media to keep its members informed and healthy and new mobile technologies to enhance staff communication and reporting. Remote diagnostic tools will also be more available to patients for common ailments like strep throat, to allow self-testing and more rapid recoveries. In the next 5 to 7 years, they see increased use of remote monitoring technology, sensors, and virtual care, as well as health analytics to enhance the nurse role in triage and care management [23]. As one interviewee put it, “…if it can be automated, it will be.”
- *Reimagining Ambulatory Design* (*RAD*) is an initiative of the Southern California Region that may spread across KP. Its goal is to design a new ambulatory care delivery model aligned to the principles of consumerism. In extensive research with members, the leads of this effort discovered that “…people wanted access to care in a much more radically different way… It has to do with much more embedding of services into the community, into the home, into work…and much more local access for simple things.” This “life-integration vision” has sparked several experiments to redesign and relocate KP clinical operations in Southern California.

#### Workforce planning and development strategies

Human resource (HR) leaders and the Coalition of Kaiser Permanente Unions (CKPU) staff report that early on the focus of WFPD was on creating consistent workforce metrics and analytics to help the regions forecast future staff and skill needs. They now view these tools as necessary but insufficient. A regional HR leader described the change:

So, at first…we forecasted membership growth, utilization, supply, turnover, retirement, we looked at the local labor markets, we connected with a university for economic analysis of the projected nursing workforce, and the fluctuations around the economy. And then we realized that most forecasting is based on the previous year, or the previous three, or the previous five years, projecting forward. But if you’re in the midst of complete transformation of how you’re providing care, how accurate are those numbers? …We need to understand what kinds of jobs (are coming); we need to understand how work is transforming. So, it really started in 2012 to 2013, (we have been) trying to get a movement towards a kind of qualitative approach to understanding change.

Key to this new approach is that it is integrated with KP’s strategic growth initiatives. As one HR leader explained, “workforce development is being driven by the business need.” Part of this emanates from the “affordability” imperative, which both HR and labor representatives agree has given finance a larger role in the company. At the same time, HR leaders describe the emerging WFPD approach as “maturing,” by which they mean that finance is one important player but that they also take into account other interests. Indeed, HR leaders view themselves as “intermediaries” who help senior leaders understand the strategic value of the workforce in the context of the drive toward labor-cost-saving solutions.

The LMP, which was further strengthened in the 2015 National Agreement, has several mechanisms that integrate labor and innovative WFPD strategies into the strategic change processes. First, for collective bargaining, they use an “interest-based approach,” rather than traditional, positional bargaining. Both sides emphasize that there is full transparency in this process—management shares information on the company’s financial situation, competitive standing, and other data related to the subjects of bargaining and labor provides insight into the affect of change on the workforce. This open exchange results in accommodation, as illustrated by the Employment and Income Security Agreement (EISA), which stipulates that any innovation or change at KP must include a plan for retaining the effected employees.

A second LMP mechanism consists of the negotiated programs to support innovation and the implication of change for the workforce*.* The national agreement delineates the mission and values of joint programs, sets aside funds, and directs LMP staff and company to consistently integrate the programs across all KP regions. Examples of these national efforts include *Total Health*, which advances wellness, health, and safety in the workplace; *unit-based teams*, which identify quality improvement and cost containment solutions at the ground level; and the *National Taft-Hartley Education and Training Trusts*, described above.

Lastly, an important characteristic of the LMP governance and planning structures is that it is holistic and aims to permeate every level of the system. In theory, every manager has a designated labor partner with whom they are encouraged to engage in strategic and operational decisions that affect the workforce. Both sides report that this works better in some regions than others, but where it does work, they say that the engagement is ongoing and includes strategic decisions that affect not only the workforce but also the future direction of the company.

Jobs for the Future, an initiative in the Southern California region, illustrates how these mechanisms work together to integrate labor and WFPD strategies into the strategic change processes at KP. The project grew from the HR leader’s intermediary strategy of showing up and intently listening at meetings related to the RAD project, a strategic change initiative aimed at redesigning ambulatory care. According to this leader, he quickly convinced the VP overseeing the project of the value of labors’ early involvement, and soon after, a LM committee was formed to explore the proposed innovation and its impact on the jobs and workers.

Rather than focus on the contentious questions of workforce impacts, the committee first set out to develop a holistic view of the redesign (new care models, technologies, facilities, etc.) in order to target the operational initiatives that would have significant impact on jobs. Though the HR lead reported that some labor and management participants fell into traditional roles and knee-jerk reactions, he observed that these positions quickly gave way as the committee became more engaged in the processes to redesign the care models and workflows.

Next, the committee developed a rigorous methodology to assess the impact on jobs and formed LM subcommittees to apply the method to the redesign of specific work areas. In the end, the committee proposed three new jobs: a roving receptionist of the future that would take on multiple roles of patient greeter/way finder/educator, a multifunctional healthcare worker that would staff new small walk in clinics and perform patent care and diagnostic functions, and a patient navigator who would facilitate the extension of care into the arena of social determinants by helping to coordinate community resources. Each of these new roles transgresses existing occupational, as well union boundaries and jurisdictions.

The difference between the new with the old approach to labor relations managing change at KP are explained by the HR leader as he reflected on this project:

The traditional way of doing it is you’re assigning labor relations people who don’t understand the operations and all the technology and innovations. They’re not included in those conversations. So they go to the bargaining table, and the labor person has only been told that there is either going to be a layoff, or a change in jobs, and we are doing this because of the need for affordability, or because we need to cater to the customer. They are like, *what!!???* So it is just kind of set up for an antagonistic type of relationship…because there hasn’t been this pre-work, conversations and joint learnings about why this change is really happening, how it will improve care. There is a big disconnect between the innovators planning this change and the bargaining with unions to implement downstream workforce implications.

Interestingly, a union representative also sees her role as an intermediary in the broader change processes at KP:

What I’m trying to do is to help facilitate the conversation. It’s really hard to make management own what they want… What classifications do you need? Where are you going to lay-off people? And where do you want to grow, right? Put it on the table, take the consequences…. And you will get (union) members that say, I am not changing… Kaiser has a lot of money; they do not need to do this… And they’re wrong, but they are human; they are afraid. (So I say) basically you’re stuck: either you learn this, or you won’t have a job… So, that’s the conversation I’m trying to facilitate. I try to get everyone to put their issues on the table and work it out…

#### Challenges

While there are many success stories in the transformation of WFPD at KP, informants also expressed concerns.

Several informants talked about the continued resistance of some business units and regional operations to the new WFPD approach. As one person explained, “the C-Suite is on board with a human capital strategy and there is a fair amount of engagement of line employees in unit-based teams, but the middle management is not fully engaged”.

While informants view the LMP as a powerful mechanism for managing the impacts of change, involving workers who are represented by unions outside the LMP and the large number of exempt employees in KP (almost half of the workforce) is challenging. As one informant put it, “So what is the governance for this work with the other half? Who sets the priorities, allocates the resources, and oversees the initiatives?”

The fluid fiscal environment and constant innovation are expanding the role of finance in strategic change and workforce decisions. Informants did not challenge the need for more fiscal control; their concern was over the episodic nature and the short-term time horizon of the financial decision-making process. As one person put it, “it does not matter if the company and the LMP have invested in a long-term strategy to fill a skills gap, finance can insist on a last minute reduction in force or a redeployment to meet fiscal targets.”

Several informants expressed the need to figure out how to bring workforce initiatives to scale and spread innovations, like the Jobs of the Future, to other regions. They believe that a deeper understanding of the knowledge, skills, and methods that underlie the emerging WFPD model might help spread innovation in KP.

### Case study 2: Montefiore Health System

The Montefiore Health System is headquartered in the Bronx, NY, and currently covers approximately 350 000 lives through a variety of value-based reimbursement relationships with commercial and government payers. Over 80 % of Montefiore’s revenue is derived from the Medicare and Medicaid programs. Its leaders describe it as an “open ecosystem” with long-standing partnerships with the community, its labor unions, community-based organizations (CBO), and local high schools and community colleges. This, we shall see, is a critical characteristic of Montefiore’s approach to workforce changes.

The organization has a long history of seeking out capitation and other forms of risk-sharing agreements. Twenty years ago, Montefiore executives formed an Integrated Provider Association (IPA), which encompassed its salaried physicians, as well as community-based, voluntary (private-practice) physicians, and approached private payers with a request to develop risk-sharing contracts. While Montefiore experienced some losses during the early days of managing these agreements, they pushed ahead, understanding that the change would take time and that returns would be realized only when there were higher volumes of covered lives. The passage of the ACA, and in particular the launching of Medicare’s Pioneer Accountable Care Organization (ACO) program, in which Montefiore was selected to be one of the original participants, opened new opportunities for value-based contracts.

From the beginning, this active pursuit of value-based contracts has been supported by a subsidiary called a Care Management Organization (CMO), which developed a robust care management infrastructure with the explicit objective of understanding and addressing the upstream determinants of health. The CMO’s approach to care coordination includes health education, linkages with social services and government benefits, health system navigation, provider communication, chronic care management and care transition management, and medication review and reconciliation. A focus on patients with high medical expense and high risk of hospital and emergency department utilization by interdisciplinary care management teams has generated savings that that are reinvested in the delivery system. Care coordination is extended beyond Montefiore’s facilities through active partnerships with community-based, voluntary physicians as well as a wide range of community service organizations.

The CMO supports this care model with a robust WFPD infrastructure that includes a comprehensive competency map for all key CMO workflows supported by a wide range of training programs to ensure employees are prepared with the required skills.

In addition to the CMO WFPD capabilities, Montefiore Human Resources (HR) and Labor and Employee Relations functions have structures and mechanisms to integrate HR as well as labor into unit-based change. For example, HR stations a HR person in every department whose role is to understand the local culture and help HR anticipate and support change. This sensing function also enables HR to ensure the engagement of labor in planned changes.

Regionally, Montefiore also has a long history of labor-management partnership through its participation and leadership in the 1199SEIU Training and Employment Fund. The fund, which was established in 1969 to provide education and job training programs for healthcare workers, is the largest joint labor-management training organization in the United States. It covers 250 000 workers (190 000 in New York City) and more than 600 employers, including hospitals, nursing homes, registered nurses (RN), and home care workers. 1199SEIU and healthcare employers jointly govern the fund and Montefiore’s Executive Vice President is on the Board of Trustees.

Since its formation in 1969, 1199SEIU has established a total of nine funded initiatives, of which Montefiore contributes to five, that cover three main areas:

- *Training and upgrading*: There are two training and upgrading funds (one specific to RN and one general) that work with Montefiore and union leaders to identify high-demand skills and occupations and develop training programs in response. It includes counseling and tutoring, adult basic education and pre-college preparation programs, and an array of college education benefits to support workers in attaining college degrees in healthcare-related occupations.
- *Job security*: An additional fund provides a safety net and rapid re-employment services for laid-off workers, who receive priority employment from hundreds of healthcare institutions in the NYC area. They also support job counseling, placement, training programs, and benefits to assist workers’ transition into a new job in healthcare.
- *Labor-management initiatives*: This fund seeks to increase worker voice in the planning and implementation of efforts to increase quality care, patient satisfaction, and operational effectiveness. It supports technical assistance on the development of joint governing structures and training in joint problem solving around quality and performance issues.

The funds are financed by collective bargaining contributions, with employers contributing 0.5 % of gross payroll to the Training and Upgrading Fund and smaller amounts to the other funds. The funds have also received over $300 million in grants to open their programs to community members and other healthcare workers who are not members of the 1199SEIU.

#### Drivers of change

The ACA’s payment reforms allowed Montefiore to leverage its experience with value-based purchasing and deepen its commitment to population health. However, New York state health policy, in particular the ambitious Delivery System Reform Incentive Payment (DSRIP) Program, a product of New York’s Medicaid Redesign Team (MRT) Waiver Amendment, is likely the greatest driver of change at Montefiore.

DSRIP will fundamentally restructure the healthcare delivery system by reinvesting in the Medicaid program, with the primary goal of reducing avoidable hospital use by 25 % over 5 years. Up to $6.42 billion dollars are allocated to this program with payouts based upon achieving predefined results in system transformation, clinical management, and population health. The entities that are responsible for creating and implementing DSRIP are Performing Provider Systems (PPS). PPS are providers that form partnerships among major public hospitals and safety net providers, with a designated lead organization for the group. There are 25 PPS across the state, with Montefiore leading one in the Hudson Valley and participating in a second PPS in the Bronx (Bronx Partners for Healthy Communities) led by St. Barnabas Hospital (SBH).

A major focus of DSRIP is to develop strategies to realign, redeploy, and retrain the healthcare workforce across the provider networks within broad regions throughout the state. DSRIP has also merged the Office of Mental Health, Office Alcoholism and Substance Abuse, and Department of Health (DOH), so there is a single regulatory structure with payment aligned. This means all community-based organizations (CBO) will begin to receive their funding from this single payer/regulator at the state level. Montefiore executives describe the program as “right-sizing” Medicaid. All care will be managed, and the number of contracts with HMOs will be dramatically reduced from 17 to 7–10 plans. Ultimately, the program’s goal is to achieve 90 % value-based payment in 5 years.

#### Change strategies

Over time, Montefiore’s leaders have realized that to make their value-based contract model work, they needed to create economies of scale. The strategy has so far resulted in the outright acquisition or other partnership arrangements with nine hospitals, several of which are in the Hudson Valley, a region that is largely exurban, dominated by solo practices, and radically different from the Bronx in terms of patient demographics. In addition, Montefiore views its engagement in DSRIP as an opportunity to expand its model to a broader continuum of care in the Bronx as well as in the Hudson Valley. Finally, it has begun to expand into new lines of business with the establishment of the Managed Long Term Care Plan (MLTCP), which may transform Montefiore into a fully integrated delivery system. The implication of these expansions is significant, both for the workforce and more broadly in terms of testing the feasibility of Montefiore’s population health model in new environments.

#### Workforce planning and development strategies

The central workforce dynamic resulting from the DSRIP rollout and Montefiore’s policy of acquisitions is that Montefiore is rapidly blurring its traditional workforce boundaries. This has multiple implications for its approach to WFPD. First, the inclusion of new facilities and regions requires HR to integrate the workforce into Montefiore’s culture, often in the context of downsizing and redeployment of staff. Second, the merging of the various social service payment schemes into one payer/regulator under DSRIP will mean that Montefiore has a direct financial interest in strengthening CBO services and, therefore, the capabilities of its workforce. Third, early discussions among partners in the PPS suggest a commitment to relocate any displaced workers from partner organizations in the PPS to avoid unemployment. This will not only intensify the imperative to expand care coordination across providers and CBO, but now extend WFPD outside the traditional boundaries of Montefiore’s employees. An HR leader described the change:

Whereas in years past we focused on our own employees and attracting top talent, now we are (also) interested in folks in the community and their future, and how to get them interested in a health care profession…We are partnering with schools, and building health care curriculums…And we have a greater focus on development and education of our community partners. We are doing more with internships and externships and volunteerism…It’s really about building the health of the community.

Montefiore’s WFPD strategies are emerging within three loosely coupled and well-resourced efforts: expansion of the CMO’s competency and training map, leveraging regional ties through its LMP, and embracing DSRIP aims to build a strong provider network. Each is closely tied to Montefiore’s strategy to build economies of scale and improve population health.

The first strategy involves the expansion of the CMO comprehensive training program to support Montefiore’s efforts to bring its care management model to scale. A core feature of this effort is a competency map that specifies what each worker needs to know and do and identifies curriculum pathways for each of the 80 clinical and non-clinical roles in the CMO. One informant shared that the map enables the CMO to scale up training and target delivery throughout the growing continuum of care.

It’s not scalable to create an education program that trains every single person here on how to arrange transportation or how to find a pharmacy that delivers. We want that to be role specific and matched to the right skill set so the training that goes with each role is then matched to what we expect people in that role to do… If we hadn’t gone to a model like that, it’s just not scalable.

The CMO model has both loose and tight elements. The loose characteristics include the placement of facilitators in the CMO units to listen and support people in developing the skills and knowledge required to continuously improve the model. There is also an educational council comprised of representatives from throughout the system that helps ensure frontline input into learning needs and evaluation of training programs. Its tightening mechanisms include standardizing some elements of training to help spread the care coordination model to the new Montefiore and the PPS partners.

The second WFPD strategy involves leveraging Montefiore’s affiliation with the 1199SEIU League Training Fund to intervene into the regional healthcare labor market to address broad workforce challenges facing the industry as a whole. For example, Montefiore, in partnership the Training and Upgrading Fund, agreed to provide a clinical site for a RN-to-BSN bridge program being offered by the City University’s Lehman College in the Bronx. This partnership brought to light Montefiore’s concerns about nursing school curricula, which are largely focused on training nurses for acute care roles and lack preparation around care coordination and population health. The partners addressed this gap in this one-time bridge program with the inclusion of a care management module. Since then, the parties have worked together to revamp the curricula to better prepare nurses for care management and care coordination careers—which include courses on the broader institutional changes in healthcare and changing care models. Montefiore and the training fund’s involvement in two regional DSRIP PPS will likely afford them an opportunity to replicate this kind of partnership with other schools of nursing and programs to train workers for other high-demand occupations.

On the internal front, though labor union relations were described as being “very collaborative” and “very well integrated into the facilities,” the degree to which the LMP is involved in Montefiore’s innovation and growth strategies is unclear. The nature of labor relations at Montefiore maybe best illustrated by the way in which CMO managers described problems redefining jobs and job titles. They essentially work hard to respect the union, but efforts to engage unions in the redefinition of jobs, as occurred in KP’s Southern California region, have not taken place.

In the union contract you have certain titles and those titles really still largely crosswalk to functions that you would have seen in a hospital or maybe in a physician’s office. But to get a new title is hard. It has to be negotiated… So what we’ve tried to do is take our functions and crosswalk them to existing titles. Our titles don’t always completely (crosswalk to the new duties)…It would be nice to have more flexibility, because it takes too long (to negotiate change).

Despite these challenges, HR leaders described their relationship with labor as being based on mutual trust and collaboration. For example, Montefiore developed training for hospital staff on Hospital-Acquired Conditions for which CMS will no longer reimburse. They partnered with 1199SEIU to roll out the program, which they believe greatly facilitated workers’ confidence that the program would be beneficial and not harmful to their interests.

The third workforce strategy involves embracing the DSRIP aims to build a strong provider network. With reduction of potentially avoidable emergency **r**oom (ER) visits and hospital admissions as end goals, the NY DSRIP stipulates that an immediate task is to “retrain the workforce for care continuum and redeploy them to ambulatory and home care.” Executives describe this challenge on several fronts. First, they report “We work across health care settings and CBO’s in the PPSs to standardize titles and competencies, and to establish criteria for determining how care will be coordinated.” They point out that this process is made particularly challenging by the vast array of ways that organizations across the PPS network have organized jobs. “Some organizations require care managers to be RNs, while others employ individuals with … a high school diploma or a GED as care managers. There is a lot of cross cutting (comparison) that we need to do.”

CMO leaders say a key challenge is ensuring that its standards are maintained as the number of organizations involved in the continuum of care expands through the DSRIP process.

There are a myriad of organizations out there that provide all kinds of services… peer groups, housing groups, mental health, substance abuse, transportation… They’re not going to be our employees… (but) we’re going to have to make decisions about (whether) we are comfortable actually turning over the responsibility for case management in a particular case.

The second area of work required by DSRIP will be to manage the relocation process. DSRIP anticipates that, over time, hospitals will reduce the number of beds or close shrink and that ambulatory-, home-, and community-based care will grow. Workers will need to be retrained to move into these new settings within PPS. The 1199SEIU League Training and Employment Fund, which spans multiple employers, will likely play a role in managing these transitions through its Job Security Fund.

#### Challenges

Despite what is largely a story of successful relationships, Montefiore informants were frank about the challenges ahead that concern them.

The first is a reflection of the need for continued maturation of the labor partnership. In particular, the lack of flexibility in renaming and redefining jobs has been an impediment to change and expansion plans. “It would be nice to have more flexibility.”

Another challenge is related to the design and use of community health workers (CHW) across the new DSRIP PPS networks. Currently, these jobs are different in their design and function, based on where the work is performed in a very broad spectrum of care coordination. Historic interests and political dynamics have in part shaped these varied roles. There are deep differences over how to integrate CHW, e.g., whether they should be hired directly into the organization, and of course, there are divergent views on which union might claim this growing cadre of workers. The question is whether the CMO’s data-driven innovation strategy will work in this highly politicalized context or whether new consultative mechanisms are also needed to successfully integrate diverse occupational roles and cultures.

The third challenge regards the spread of the model to the Hudson Valley. Currently, Montefiore’s relationship with its newly acquired facilities in the region is largely financial—but ensuring institutional stability will require Montefiore to transport its care coordination and community-based approach. This model is in part reliant on a large system that can move workers affected by change in one facility to new roles and locations in the expanding continuum of care. It remains to be seen whether there are the workforce relationships and mechanisms that will facilitate such processes in this suburban and exurban area of the state.

## Discussion

Though KP and Montefiore are very different systems, each mounting a different strategic response to the ACA, they share a common understanding of the centrality of the workforce in any delivery system change process. This is reflected in a series of common themes that emerged in relation to our central study questions: how are these systems determining what changes are needed, and how they are implementing change in practice? Below, we identify five broad themes present in both systems and discuss in the context of the theory of LCS. We then extrapolate the principles in each that may be relevant to other health systems and to broader issues of workforce policy and practice.

### Core values and a centralized vision

The first theme common to these case studies is that both organizations have a set of strong core values and a centralized vision with regard to their goals. At KP, the history of pre-paid, member-based service has instilled a core value for health prevention, while its roots as an innovator in the delivery of comprehensive medical services to workers and their families contributed to KP’s vision for continuous innovation and healthy workplaces. These values and vision appear to be one explanation for KP’s extensive investment in the LMP and the many LM programs aimed at improving working conditions and making KP an employer of choice. Extensive engagement of labor in change decisions, coupled with the integration of innovation units into the change projects, helps to ensure that these values and vision are key factors in determining the needed change in KP. More recently, participation in the Health Exchanges has led to the adoption of additional values centered on the ideas of consumer convenience and affordability. These new values are also informing the current cycle of innovation and change in the company.

At Montefiore, the core value of population health not only directs internal change, it underlies its efforts to build extensive external partnerships aimed at improving the entire continuum of care in the region. Regardless of whether WFPD is focused on current employees or the external pipeline of people who need jobs, Montefiore informants view these investments as part and parcel of a population health strategy. An HR leader summarized the viewpoint: “…we believe [these external WFPD programs] are good for us as an organization.” In addition, Montefiore’s centralized vision of socially oriented care links and integrates many locally driven innovations and care models to the overall system. “…Every facility [in the Montefiore Health System] has its own culture, but the core is… our vision and our values.” These values and vision are embedded in the formal and informal processes that drive care and change at Montefiore. “If they (a newly acquired facility) are following the process, the culture starts changing; there is no other way.”

Weick [11] and Burke [17] argue that large-scale, institutional change, like that occurring in healthcare today, requires a high degree of cooperation that is difficult to achieve among the many semi-autonomous subunits and organizations in LCS. Burke suggests that shared values help remind people why the system exists in the first place, while a centralized vision contributes focus within the dynamic complexity of LCS.

In both cases, we see that their historical and cultural context is key to understanding how they integrate WFPD activities into ongoing change processes. The emerging principle, then, is that *the situation* determines what type of adaptive WFPD is possible in the first place. This means that WFPD is not just a technical exercise; it must also appraise the political, economic, cultural, and social dynamics within specific contexts in which healthcare takes place [13]. To be effective, the process must consider the multiplicity of values that drive healthcare and WFPD decisions [13].

### Transparency and early dialogue

The second theme that emerged in both cases is the commitment to transparency with regard to the goals and criteria for making decisions about changes and to an early dialogue with stakeholders, in particular labor, around the best way to organize the change. In both systems, we see an institutional commitment to early collaboration with labor and other key partners throughout the change process.

In KP, the national agreement and the investment in the LMP have resulted in a highly integrated system of corporate governance that involves labor in strategic decisions on every level of the company, from the UBT to national strategic planning efforts. The sharing of sensitive corporate information and performance data is essential to making these efforts work.

The extent to which labor is involved in determining internal change in Montefiore is unclear, though HR leaders did talk about the importance of early dialogue with labor about planned changes: “…we contact them early so that they do not hear about things late.” According to an HR lead, this early consultation results in labor buy-in, which in turn provides employees with the assurances they need to engage in change.

Greater emphasis on transparency and early dialogue between Montefiore and 1199SEIU, its largest union, was observed in external efforts to close gaps in the labor market and in their mutual engagement in the DSRIP planning process. The expansion of the one-time nurse bridge program to create a new curriculum to prepare nurses for care coordination roles is an example of how joint leadership resulted in improvements to the WFPD infrastructure in the region.

The theory of LCS suggests that transparency and early dialogue are highly functional change mechanisms, because they open the process to many different interests and vantage points required for sense making [25]. In addition, these mechanisms create shared leadership, which is more effective than hierarchical leadership when seeking to tighten connections within a LCS [17].

An emerging principle then is that WFPD is *integrated* with strategic and operational planning processes. Beekun and Glick [16] define integration as a process for achieving unity of effort among various subsystems in the accomplishment of the organization’s tasks and goals. Moreover, from a change perspective, efforts to integrate are seen as boundary defining and boundary spanning, which is a political process that requires ongoing negotiation and mutual adjustment [17]. With these concepts in mind, this principle suggests that WFPD is a dynamic process of negotiation and mutual adjustment among semi-autonomous subunits in a LCS that seeks to integrate the workforce into the change processes within firms, as well as, as we shall discuss below, to align internal change with the system-wide skill formation goals and activities of WFPD institutions.

### Innovations to workflow

The third theme is changes often emanates from innovations to workflow that emerge from an analysis at the unit level and then take into account competing interests across the system. This is in contrast to change defined based on existing jobs and organizational structure or simply an analysis of who currently does what.

For example, KP’s UBT engage in the process on an ongoing basis. In Southern California, efforts to massively revamp ambulatory care based on the principles of consumerism began at a central level with a complete rethink of consumers’ wants and then engaged stakeholders in a discussion about how and where work is carried out, as well as who does what.

The innovation model in Montefiore also starts with an analysis of the optimum work design at the unit level, as opposed to the current workflows and job structures. The CMO competency map then uses the local analysis to build a whole-system approach to WFPD. It identifies the range of knowledge and skills that are required for coordination across the continuum of care, and it delineates what every occupation group needs to know and do to support the care model. This tool ensures that the required expertise is available across the entire system, while it also enables the customization of curriculum pathways for each role and individual in the CMO.

There are several emerging principles here. The first related once again to integration, as discussed above. But in addition, we see principles of both a holistic approach and an approach that is adaptive to changing demand.

The *holistic* principle implies the consideration of the whole system of professions and occupations, as opposed to each profession having its own distinct role, training structure, and regulatory mechanisms. Dussault and Dubois posit that a traditional siloed approach in healthcare hinders the implementation of policy and complicates the change process, particularly when new, multidisciplinary models that require a high degree of interdependence among many different professions are required [13].

A related principle is that adaptive WFPD must be *responsive to changing demand*. Both systems have concluded that the traditional linear approach to WFPD is necessary but not sufficient. Their adaptive approaches begin with a focus on the demand for healthcare and try to account for the macro shifts and trends as well as the internal political dynamics affecting the health system and its workforce [13]. In the complex setting of healthcare today, this requires a highly participative decision approach that accounts for many perspectives that is also supported by accurate, robust, and accessible data that can account for the large and growing number of variables that affect the demand for care and the supply of the workforce [10, 13]. New methods are also required that can utilize the new so-called big data systems to model the efficacy of possible care models and WFPD scenarios [10].

### New patterns of coupling

The fourth theme is the new patterns of coupling, including both tightening and loosening of the alignment of each company’s component parts. These changes are consistent with the proposition of Bechun and Glick, who argue that institutional changes can set into motion new patterns of coupling within organizations as they respond to the changing environment. They also claim that the strategies used to foster new patterns of coupling will reflect the organizations’ traditional approach to implementing change.

We have seen that, historically, KP grew as a loosely coupled network of providers (Permanente Group) and an insurer (Kaiser Foundation Health Plan), across seven semi-autonomous regions. Recent efforts to streamline administrative systems through “One KP,” as well as HR’s work to create centralized skill standards and training, reflect an attempt to cut costs and to create a common corporate identity by tightening connections. In addition, the influx of new individual members is pushing KP to both loosen and realign their historic workflows and communication patterns by relocating care and consolidating roles to improve care and make it more accessible. Lastly, we see how technology continues to play an important role in meeting long-held objectives to tighten the connection between KP and its members, as well as, they hope, to improve the quality of care.

Similarly, Montefiore’s historic strategy to promote value-based contracting led them to extend their care model by tightening their connections throughout a loosely coupled network of providers, and this approach continues to grow as they expand into new regions. Once connections are made, CMO takes the lead in tightening efforts by identifying the parts of the system where outcomes are weak and costs are high and then turns the focus to the redesign of work, followed by training, both initial and continuous. Now, with the expansion of the system into new regions, and the new relationships with external providers and community-based organizations that are being formalized through DSRIP, the CMO is poised to integrate its approach with external partners.

The emerging principle here is again *integration*, not just with regard to internal realignment but with external relationships as well. This is particularly striking in the case of Montefiore, where their new patterns of integration are aligning internal change with external partnerships.

### Maturing the WFPD model through intermediary functions

Both systems work hard to continuously mature their approach to WFPD. Indeed, Burke anticipated that as LCS grew more commonplace in business and society, change agents would need deeper knowledge of the dynamics of LCS and more complex change strategies to enable both the tightening as well as the loosing of ties throughout the system. Change agents know how to tighten, according to Burke, but few can discern the need to loosen and then effectively intervene.

Informants in both cases were quite articulate about their WFPD model and the need to improve and expand it beyond the traditional approach. Intermediary structures form the structural basis for the WFPD model in both cases. Intermediaries, according to Giloth, broker and integrate a variety of interests and resources to enact WFPD in local settings [26]. As seen in both cases, these intermediaries devote a great amount of “face time and linguistic work” to help people make sense of the ambiguity brought about by the unpredictable structures within LCS [25].

The intermediary partnership in KP is made up of a loosely coupled network of staff housed in a variety of support units who “show up” at important corporate innovation meetings and establish a presence in the change process. In addition to showing up, informants talked about a variety of intermediary strategies, such as aggressive engagement, deep listening, and accommodation, that they utilize to help HR and the LM to “translate the workforce and labor piece” of the change. Thus, in KP, the intermediary strategies provide a valuable sense-making function that help the parties respond to change. One informant claimed that this intermediary, sense-making approach “…is core to how you transform workforce planning.”

Intermediary partnerships also provide sense-making functions throughout Montefiore. The HR Business Partners also show up at business meetings and they link HR processes to change occurring in operational units. The CMO’s Education Council and frontline facilitators are listening mechanisms that align training with change occurring in the system. Lastly, in the context of DSRIP, Montefiore participates in workforce planning in the Bronx and the Hudson Valley PPS. In the Hudson Valley, they are engaging provider HR and operations staff in a preliminarily needs assessment to help make sense of future staffing needs and to identify gaps and resources. This was described as an analytical process to develop a strategy to close the gaps required to make each new role successful.

A major emerging principle regarding this theme is that decisions are being made through a *process of consensus building* that includes workers and are accommodative of the needs, interests, and preferences of participating groups. Again, WFPD is both a political and technical exercise [13], and as such, it calls for a process of exchange, negotiation, and mutual adjustment [11] among a diverse range of stakeholders. Consensus is necessarily achieved through processes of accommodation to the needs, interests, and preferences of the client. According to Burke [17] change agents must accept that they cannot fully understand and appreciate the client’s deep situational knowledge and approach each setting with curiosity. This openness promotes learning and shared ownership of WFPD problems, activities, and programs.

In addition, we see that WFPD is a process that is *continuous and iterative*. Dussault and Dubois suggest that the historic system of professional dominance in healthcare calls for an ongoing process (continuous) of adjustment (iterative design) that can attend to population needs as well as the changing expectations and roles of the healthcare workforce [13]. Further, Weick and Burke suggest the ambiguous and complex inter-relationships in LCS require an improvisational change process that connects past experience and knowledge (continuity) to present novelty through tinkering (incremental change) [17, 27]. Achieving this approach requires an eclectic mix of listening, practice, modeling, the ability to recognize the partial relevance of previous experience, and a high confidence in skill to deal with non-routine events [11].

Finally, WFPD is *generative*, resulting in new resources and capacity for innovation. We see in both cases that WFPD requires institutional capacity and the investment of time and money in sense making and structuring activities [13]. Both cases demonstrate how the interest in listening and accommodation has implications for intermediary WFPD structures and resources. And resource allocation for these functions is significant, in particular for the LMP at KP. Both organizations are also recruiting top talent in workforce development and place a high value on the expertise of their employees in this area.

### An emerging framework

The seven principles emerging from these case studies, when considered synergistically, help provide a framework for thinking about adaptive WFPD in specific contexts. Table 1 synthesizes our findings for this purpose.

**Table 1:** Emerging themes and their related principles

Common themes in case studies	Related theoretical principles
1. Strong core values and vision	1. Historically and culturally *situated*
2. Transparency and early dialogue with labor and other stakeholders	2. *Integrated*, internally and externally, with strategic and operational process
3. Innovations in workflow	(2) Integrated 3. Changes are *holistic* 4. Changes are *adaptive* and based on data about changing demand
4. New patterns of coupling	(2) Integrated
5. Maturing the WFPD model through intermediary functions	5. A process on ongoing *consensus* building 6. *Continuous* and iterative 7. *Generative* of new investment in the function

This emerging framework is consistent with the theory of LCS and resonate strongly with the critique offered by Dussault and Dubois of human resource planning in the healthcare sector. We would suggest that others could adopt these propositions to think about WFPD in new and innovative ways

## Conclusions

This comparative case study analysis suggests that the old way of doing WFPD by estimating the workforce needs within the confines of an institutional setting is giving way to new adaptive approaches. Institutional settings in the context of a post-ACA landscape are simply too complex and fast paced for the old approach to work. Both KP and Montefiore understand that the only way to do WFPD in periods of rapid transformation is to engage—to listen and interpret what is happening from a workforce perspective. This process requires developing an institutional capacity for sense making [11] across the organization, achieved through a continuous process of engaging, listening, and organizing.

WFPD is therefore no longer a centralized function at KP and Montefiore. Control mechanisms have been replaced with strategies to integrate various stakeholders and units across a broad continuum of WFPD activities and programs. The new approach to WFPD is aligned with strategic growth plans and is integrated with labor, employment relations, innovations teams, and local change initiatives. Both systems demonstrate a concern for the impact of change on their workforce and have made large political as well as financial commitments to avoid outsourcing and layoffs.

We find that a series of new change principles defined by theorists as suitable for improving the functioning of LCS [11, 17] and aligned with the adaptive WFPD model [10, 13] are present in both organizations. The principles include WFPD that is (1) situated in a set of core values that have emerged from specific historic and cultural contexts; (2) integrated, both internally and externally; 3) focused on a whole-system perspective; 4) responsive to changing demand; 5) based on consensus building, that is (6) continuous; and (7) generative and requires real and continued investment.

The effective implementation of these principles in these two major health systems has given rise to a pattern of reciprocal interdependence and mutual adjustment among the diverse range of actors across the WFPD ecosystem. This new form of coordinating WFPD across the system is both enabled by and helps to foster a form of knowledge-based action and a pattern of thinking that is slow, deliberate, and explicit—and is more aligned with the complexity of health workforce changes in a post-ACA environment.

These findings may be relevant to a range of other healthcare organizations. While the payment reforms that are spurring workforce transformations may be different for public systems, like the Veterans Health Administration in the United States, to the extent that they are embracing any major system changes, these WFPD principles would be applicable. In other words, the principles are about managing change in complex organizations, not about the specifics of the changes.

The findings may also hold meaning for macro-level workforce policies at the state and federal governments. Technocratic WFPD at these levels is also likely to be insufficient during periods of large-scale system transformation. Traditional policy levers, such as scope of practice regulation, education and training curriculum and degrees, and professional codes, have not been responsive to the needs of LCS, because they are designed to ensure uniformity in roles and job structures across the broader healthcare system.

If today, we are likely to see less uniformity in our distributed, free-market healthcare system as it continues to innovate and multiply new models of care, then WFPD at both the organizational and the public policy levels must also go beyond data analysis and engage in a political process of spanning traditional boundaries, listening to diverse interests, and building consensus. It requires new intermediary structures, and it must be generative of new resources and new talent. It also requires building the political and technical skills of WFPD professionals and empowering them to challenge old practices and ways of thinking about workforce issues and problems [13] and address the structural and financial gaps in the skills formation continuum.

## Abbreviations

ACA: Affordable Care Act
ACO: Accountable Care Organization
CBO: Community-based organizations
CHW: Community health workers
CKPU: Coalition of Kaiser Permanente Unions
CMO: Care Management Organization
DOH: Department of Health
DSRIP: Delivery System Reform Incentive Payment Program
EISA: Employment and Income Security Agreement
ER: Emergency room
HIT: Health information technologies
HMO: Health Maintenance Organization
HR: Human resources
IT: Information technologies
KP: Kaiser Permanente
LCS: Loosely coupled systems
LMP: Labor-management partnerships
MRT Waiver Amendment: Medicaid Redesign Team Waiver Amendment
PMG: Permanente Medical Group
PPS: Performing Provider Systems
RAD: Reimagining Ambulatory Design
RN: Registered nurse
UBT: Unit-based teams
WFPD: Workforce planning and development
